# Different Patterns of Bacterial Species and Antibiotic Susceptibility in Diabetic Foot Syndrome with and without Coexistent Ischemia

**DOI:** 10.1155/2021/9947233

**Published:** 2021-04-27

**Authors:** Rafał Małecki, Kamil Klimas, Aleksandra Kujawa

**Affiliations:** ^1^Department of Angiology, Systemic Hypertension and Diabetology, Wrocław Medical University, Borowska 213, 50-556 Wrocław, Poland; ^2^University Hospital of Jan Mikulicz-Radecki, Borowska 213, 50-556 Wrocław, Poland

## Abstract

**Aims:**

Infection in diabetic foot syndrome (DFS) represents serious medical problem, and the annual risk of DFS in diabetic patients is 2.5%. More than half of the patients with DFS have symptoms of extremity ischemia (peripheral arterial disease (PAD)). The aim of the present study was to analyze the frequency of particular bacterial strains in people with DFS, analyze the impact of arterial ischemia on the occurrence of a given pathogen, and evaluate the antibacterial treatment based on the results of bacterial culture.

**Methods:**

The analysis included 844 bacterial strains obtained from 291 patients with DFS hospitalized in the Department of Angiology in years 2016–2019.

**Results:**

The most common isolates were *Staphylococcus aureus*, *Enterococcus faecalis*, *Enterobacter cloacae*, *Pseudomonas aeruginosa*, and *Acinetobacter baumannii*. Nearly 20% of the species were found to have at least one resistance mechanism. In patients with PAD, Gram-negative species were isolated more commonly than in people without PAD. The most useful drugs in DFS in hospitalized patients are penicillins with beta-lactamase inhibitors, 3rd- to 5th-generation cephalosporins (with many exceptions), carbapenems, aminoglycosides, and tigecycline.

**Conclusions:**

Bacterial strains isolated from ischemic DFS are more resistant to commonly used antibacterial agents, i.e., penicillins (including penicillins with beta-lactamase inhibitors), cephalosporins (except for the 4^th^ and 5^th^ generations), glycopeptides, and linezolid. When planning treatment of hospitalized patients with DFS, the presence of ischemia in DFS should always be taken into consideration. It determines the occurrence of particular bacterial species and the choice of antibacterial agent and may determine the rate of treatment success.

## 1. Introduction

Diabetes mellitus is a social disease with the prevalence more than 5% that exerts a heavy burden on the healthcare system. One of the most common chronic complications of diabetes mellitus is diabetic foot syndrome (DFS)—defined as an infection, ulceration, and/or destruction of the foot in patients with diabetic neuropathy or peripheral arterial disease (PAD). The estimated global prevalence of DFS is 6.3% among patients with this disease [1]; it is also known that 20% of all diabetic patients require hospitalization because of DFS, and the annual risk of developing this complication is 2.5% [2].

One of the most serious problems faced by physicians treating patients with DFS is an introduction of appropriate empiric antibacterial therapy before the results of microbiological culture are collected and antibiogram is available. The aim of the present study was to analyze the frequency of particular bacterial strains in people with DFS, analyze the impact of arterial ischemia on the occurrence of a given pathogen, and evaluate the antibacterial treatment in this group of patients, taking into account the presence of PAD.

## 2. Material and Methods

The analysis included 291 patients hospitalized in the Angiology Clinic in the years 2016–2019 with a diagnosis of DFS with infection. According to IDSA guidelines, infection was diagnosed if two symptoms of inflammation (erythema, warmth, tenderness, pain, and induration) or purulent secretion were found [3]. In all the patients, a microbiological culture was performed using properly obtained material from ulceration (wound). The material was taken after rinsing the wound with 0.9% NaCl solution from the most profound obtainable tissues; tissue aspirates and material collected during surgical debridement or amputation were also cultured. The disk-diffusion method with paper discs impregnated with antibiotics at a specific concentration was used to determine the susceptibility of microorganisms to antibiotics and chemotherapeutics. The detailed protocol of the testing can be found in the literature [4]. The size of the inhibition zone around the disc indicates the susceptibility of the particular bacterial strain to the analyzed antibacterial agent.

The patients were classified as having ischemic DFS (if peripheral arterial disease (PAD) was present, irrespective of the presence of polyneuropathy) or as having nonischemic DFS (if peripheral arterial disease was absent and there was polyneuropathy). Polyneuropathy was diagnosed based on the patient's history and the results of physical examination including assessment of temperature (using Tip-Therm), touch (10 g monofilament), pinprick, vibration (128 Hz tuning fork), and reflexes (Achilles tendon reflex and knee reflex) [5]. If the results of neurological examination were not conclusive, electromyography and electroneurography were performed. The diagnosis of peripheral arterial disease (PAD) was established according to the current guidelines by means of accessory examinations, i.e., ankle-brachial index (ABI), Doppler ultrasound of the extremity vessels, computed tomography angiography, angio-MRI, or arteriography [6].

The obtained results were analyzed statistically. In the case of normally distributed variables (identified by the Shapiro-Wilk test) and homogeneity of variance (confirmed by the Levene test), differences between groups were determined using Student's *t*-test. Alternatively, in the case of nonnormal distributed variables, the Mann–Whitney *U* test was applied. Intergroup differences in the percentage distributions of dichotomous variables were analyzed with Pearson's *χ*^2^ test. *p* value < 0.05 was considered statistically significant. All calculations were conducted with the Statistica version 13.3 (TIBCO Software Inc.).

## 3. Results

The analysis included 844 bacterial strains obtained from 291 patients with DFS (183 males and 108 females) at the mean age of 65.38 (±11.80) years. One bacteria strain was obtained only in 99 people (34.02%), 2 strains in 66 people (22.68%), 3 strains in 44 people (15.12%), and more than 3 strains in 82 cases (28.18%). Gram-positive (no = 426, 50.47%) and Gram-negative strains (no = 418, 49.53%) occurred almost equally often. 52 strains of anaerobic bacteria (6.16%) were isolated.

The most common isolated bacteria were *Staphylococcus aureus* (no = 211, 25.00%), *Enterococcus faecalis* (no = 96, 11.37%), *Enterobacter cloacae* (no = 66, 7.82%), *Pseudomonas aeruginosa* (no = 58, 6.87%), and *Acinetobacter baumannii* (no = 54, 6.40%). All isolated strains are presented in Table 1, in patients with nonischemic DFS in Table 2, and in patients with ischemic DFS in Table 3. As many as 162 isolated strains (19.19%) were found to have at least one resistance mechanism; the most important types of resistance and its percentage shared in particular bacteria are presented in Table 4.

Relationships between the results of laboratory test and the etiological factor were nonsignificant, with the exception of the percentage of glycated hemoglobin A1c (HbA1c). HbA1c was higher in infections with *E. faecalis* than in other bacteria (9.26 vs. 8.68%, *p* = 0.02245); a similar relationship was found for *A. baumannii* (9.31 vs. 8.72%, *p* = 0.04768). On the other hand, in people with *E. cloacae* infection, a lower level of HbA1c was observed compared to other bacteria (8.13 vs. 8.80%, *p* = 0.01718); a similar trend was shown regarding *P. aeruginosa* infection (7.96 vs. 8.81%, *p* = 0.00383).

**Figure 1:**
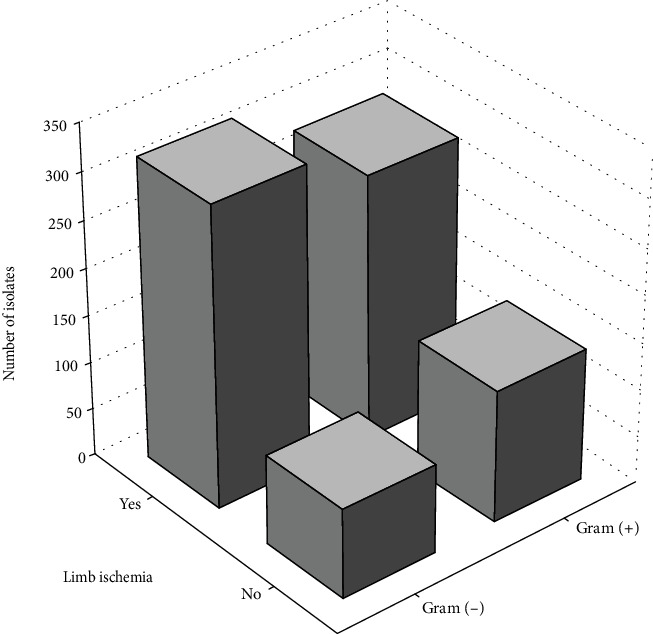
The proportion of Gram-positive and Gram-negative bacteria in patients with and without coexistent peripheral arterial disease (extremity ischemia).

369 isolates (43.72%) were obtained from people with neuropathic-ischemic DFS, 239 (28.32%) from ischemic DFS, and 236 (27.96%) from neuropathic DFS. In patients with PAD, Gram-negative species were isolated more commonly than in people with normal extremity perfusion (53.18 vs. 40.25%, *p* = 0.00077) (Figure 1), whilst anaerobes were cultured equally often in both groups. In patients with PAD, *E. cloacae* was isolated almost twice as often as in patients with normal extremity perfusion (8.88 vs. 4.66%); in other cases, there were no significant differences in regard to main etiological factors.

Carbapenems, especially meropenem, tigecycline, and aminoglycosides turned out to be the most useful antibiotics in monotherapy followed by 4^th^ and 5^th^ generations of cephalosporins and penicillins with beta-lactamase inhibitors. Their empiric usefulness, however, partially depends on the type of DFS (ischemic or nonischemic). This relationship is particularly pronounced in the case of amoxicillin with clavulanate, 1^st^-generation cephalosporins, and glyco- and lipopeptides (more useful in the neuropathic DFS), as well as ceftazidime, aztreonam, levofloxacin, moxifloxacin, and colistin (more useful in DFS). The differences in the utility of antibacterial agents in particular types of DFS are presented in Table 5. Noteworthily, low sensitivity of bacterial strains to metronidazole, macrolides, and clindamycin was found in all patients.

Patients included in the study were hospitalized, and according to the current guidelines in such circumstance, the empiric treatment should consist of at least two antibacterial agents. The most common treatment regimens cited in the literature and their usefulness in patients with ischemic and nonischemic DFS were analyzed (Table 6). The combination of amoxicillin/clavulanate with vancomycin turned out to be less useful by almost half in people with nonischemic DFS than in patients with coexistent PAD (a similar relationship was also observed for piperacillin/tazobactam and vancomycin); the opposite correlation was found for the combination of carbapenems with vancomycin. Fluoroquinolones together with clindamycin, ceftazidime, and metronidazole showed unacceptably low utility, and the treatment regimen based on ceftazidime with clindamycin was only suitable in 52%.

An attempt was made to establish acceptable and applicable regimens of empiric antibiotic therapy, excluding antibiotics with serious side effects (e.g., colistin and vancomycin), used only in the case of resistance to other drugs and after receiving the results of microbiological culture (e.g., carbapenems) and expensive, hardly available antibiotics (e.g., 4^th^- and 5^th^-generation cephalosporins, linezolid, and tigecycline). The results of the analysis are presented in Table 6.

## 4. Discussion

As in our previous study [7], Gram-positive and Gram-negative strains were isolated with almost the same frequency. It is considered that infections with Gram-positive bacteria are more common in Western communities, whilst Gram-negative bacteria are more common in Eastern communities [8]. However, this explanation seems to be unsatisfactory with respect to the high percentage of Gram-negative bacteria observed in our group. A possible explanation is that the analyzed population included hospitalized patients, previously treated in various hospital wards, with more severe infection involving more than one bacterial strain, commonly with coexistent PAD. Because the Department of Angiology is a part of the general health system, the study group most probably represents the population of hospitalized patients in general.

Despite a similar distribution of Gram-positive and Gram-negative species, the prevalence of particular bacteria is different compared to our study from 2014. The most common isolate in the aforementioned study had been *Enterococcus faecalis* (16.08%), which in the present analysis has taken the second position (11.37%), as nearly one-fourth of all infections are caused by *Staphylococcus aureus* that predominate in the study. *Enterobacter cloacae* was at third place, which may be alarming because of the high tendency of this species to produce mechanisms of antibiotic resistance [9]. *Pseudomonas aeruginosa* continues to be the fourth most frequently isolated pathogen among patients with DFS. The fifth most often isolated pathogen is *Acinetobacter baumannii* (6.40% compared to 2.01% in 2014), which is concerning due to the evidently hospital origin of this strain and its significant resistance to antibiotics [10]. Noteworthily, the low frequency of *Streptococcus* bacteria can partially result from a use of beta-lactam antibiotics as first-line drugs in the general population.

The common occurrence of strains resistant to antibiotics is especially problematic, as many as 20% isolates have at least one resistance mechanism, and the MDR strain accounted for 70% of isolated *Acinetobacter baumannii* (distribution similar to observed in other centers [11]). The resistance of one-fifth of all bacteria in the population with DFS has serious consequences for treatment effectiveness, since standard empiric with antibacterial agents cannot be successful in more than 80% of cases.

In the present analysis, the susceptibility of bacteria to antibiotics was analyzed in relation to algorithms presented in available guidelines [12, 13]. Although monotherapy with meropenem covers 82% of isolated strains, in case of other antibacterial agents, this proportion does not exceed 75% (tigecycline) and 68% (aminoglycosides). Penicillins with beta-lactamase inhibitor were suitable in more than 50% of cases, similar to cephalosporins of 4^th^ generation and 5^th^ generation (with exception of ceftalozane). Some 3^rd^-generation cephalosporins (ceftriaxone, cefotaxime) were useful in less than 50% of isolates.

The guidelines in severe infections usually recommend intravenous ciprofloxacin with clindamycin (only 46% accuracy in our study), amoxicillin/clavulanate with vancomycin (62%), piperacillin/tazobactam with vancomycin (87%), amoxicillin/clavulanate with cotrimoxazole (73%), ciprofloxacin with linezolid (64%), and moxifloxacin with linezolid (59%). In the present study, a high proportion of susceptible bacteria have been found in relation to amoxicillin/clavulanate with amikacin (83%) and ceftriaxone with amikacin (77%); more available and cheaper cefuroxime with amikacin has the accuracy of 76%. We also proved low usefulness of some groups of drugs in DFS, i.e., fluoroquinolones and macrolides. Despite the special role of clindamycin and metronidazole in anaerobic infection, their accuracy in this purpose is limited (58% for clindamycin and 54% for metronidazole), compared to amoxicillin/clavulanate (90%).

PAD is an important factor affecting prognosis in patients with DFS. Various analyses have shown different rates of PAD in people with diabetes, ranging from 49% in the EURODIALE study [14] to about 60% in analysis involving smaller populations [15]; however, some researchers postulate that this proportion may be higher [16]. In the analyzed population, the incidence of PAD was 72.02% (including patients with ischemic diabetic foot without neuropathy and mixed, ischemic-neuropathic DFS). In meta-analysis involving over 50,000 patients with DFS, the presence of PAD was associated with two times higher risk of major limb amputation [17]. Nevertheless, data on diversity of particular pathogens and their susceptibility to antibiotics in patients with diabetes and PAS is scarce.

In the present study, it was found that Gram-negative bacteria occurs about 1/4 more frequently in ischemic compared to nonischemic DFS, which may result in a different sensitivity to commonly used groups of antibacterial agents. Moreover, it was shown that bacterial strains isolated from ischemic feet are more resistant to the most commonly used groups of antibiotics, i.e., penicillins (including combinations with their inhibitors), cephalosporins (except for the 4th and 5th generations), glycopeptides, and linezolid. Although the shift towards Gram-negative bacteria is well known in the literature for extremity ischemic ulcers [18], it is uncommonly taken into consideration in the context of DFS.

We can also speculate that differences in isolate patterns between ischemic and nonischemic DFS are not only a consequence of the higher morbidity and more frequent contact with health care but also may result from different local environments of neuropathic and ischemic ulcers. Indeed, in a typical diabetic foot, the infection is driven by neuropathy and its sequelae, hyperglycemia, and probably dysfunction of the immune system [19]. Ischemia may additionally favor the development of Gram-negative bacteria (e.g., there are reports of increased invasiveness of Gram-negative bacteria, e.g., in people with anemia) [20].

There are some limitations in our analysis. Undoubtedly, the effectiveness of a given chemotherapeutic agent is determined by its clinical effect, not by the result of the antibiogram, which comprises only one possible variable. Besides drug availability and compliance, the accuracy of therapy is also determined by other factors not included in the analysis, e.g., tissue penetration of antibacterial agents. For example, it is known that vancomycin is characterized by poor tissue penetration, as opposed to aminoglycosides (moderate penetration) or cotrimoxazole (good penetration) [21]; obviously, in DFS therapy, using drugs with good penetration is preferred. Notwithstanding, the result of antibiogram is always the first step in choosing appropriate therapy and reducing the number of modalities to susceptible medications.

## 5. Conclusions

(1) The most common isolated bacteria in patients with DFS were *Staphylococcus aureus*, *Enterococcus faecalis*, *Enterobacter cloacae*, *Pseudomonas aeruginosa*, and *Acinetobacter baumannii*. In patients with PAD and DFS, Gram-negative species were isolated more commonly than in people with neuropathic DFS, whilst anaerobes were cultured equally often in both groups. In patients with PAD, *E. cloacae* was isolated almost twice as often as in patients without PAD

(2) Including all analyzed patients with DFS, monotherapy with meropenem covers 82% of isolated strains, but in the case of other antibacterial agents, this proportion does not exceed 75% (tigecycline) and 68% (aminoglycosides). Penicillins with beta-lactamase inhibitor were useful in more than 50% of cases, similar to cephalosporins of 4^th^ generation and 5^th^ generation (with exception of ceftalozane). Some 3^rd^-generation cephalosporins (ceftriaxone, cefotaxime) were suitable in less than 50% of isolates. Contrarily, clindamycin, metronidazole, and macrolides are definitely less useful and should not be used in the treatment of DFS

(3) Gram-negative bacteria occur about 1/4 more frequently in ischemic compared to nonischemic DFS, which may result in a different sensitivity to commonly used groups of antibacterial agents. Moreover, bacterial strains isolated from ischemic feet are more resistant to commonly used antibacterial agents, i.e., penicillins (including penicillins with beta-lactamase inhibitors), cephalosporins (except for the 4^th^ and 5^th^ generations), glycopeptides, and linezolid. In ischemic DFS, merely aztreonam, carbapenems, and fluoroquinolones (a high proportion of resistant strains) appear to be more useful

(4) The most potent combinations of antibacterial agents were carbapenems with vancomycin, piperacillin/tazobactam with vancomycin, ciprofloxacin with linezolid, and moxifloxacin with linezolid. The combinations of fluoroquinolones with clindamycin or ceftazidime with metronidazole showed unacceptably low efficacy. The therapy based on ceftazidime with clindamycin was accurate only in half of the isolates

## Figures and Tables

**Table 1:** Number of particular bacterial isolates in all patients with diabetic foot syndrome.

	Number of isolates	Percent
*Staphylococcus aureus*	211	25.00
*Enterococcus faecalis*	96	11.37
*Enterobacter cloacae*	66	7.82
*Pseudomonas aeruginosa*	58	6.87
*Acinetobacter baumannii*	54	6.40
*Klebsiella pneumoniae*	50	5.92
*Escherichia coli*	44	5.21
*Proteus mirabilis*	31	3.67
*Streptococcus agalactiae*	24	2.84
*Proteus* spp.	19	2.25
*Enterococcus faecium*	17	2.01
*Morganella morganii*	17	2.01
*Finegoldia magna*	12	1.42
*Enterobacter aerogenes*	9	1.07
*Klebsiella oxytoca*	9	1.07
*Streptococcus mitis*	9	1.07
*Stenotrophomonas maltophilia*	7	0.83
*Veillonella* spp.	6	0.71
*Anaerococcus prevotii*	5	0.59
*Citrobacter freundii*	5	0.59
*Peptoniphilus asaccharolyticus*	5	0.59
*Streptococcus dysgalactiae*	5	0.59
*Bacteroides fragilis*	4	0.47
*Citrobacter braakii*	4	0.47
*Proteus vulgaris*	4	0.47
*Proteus penneri*	4	0.47
*Streptococcus pyogenes*	4	0.47
*Streptococcus constellatus*	4	0.47
*Clostridium sporogenes*	3	0.36
*Prevotella* spp.	3	0.36
*Providencia rettgeri*	3	0.36
*Serratia marcescens*	3	0.36
*Citrobacter koseri*	3	0.36
*Acinetobacter lwoffii*	2	0.24
*Actinomyces naeslundii*	2	0.24
*Bacteroides distasonis*	2	0.24
*Bifidobacterium* spp.	2	0.24
*Citrobacter youngae*	2	0.24
*Clostridium innocuum*	2	0.24
*Clostridium novyi*	2	0.24
*Corynebacterium striatum*	2	0.24
*Lactobacillus fermentum*	2	0.24
*Peptostreptococcus* spp.	2	0.24
*Prevotella melaninogenica*	2	0.24
*Propionibacterium acnes*	2	0.24
*Staphylococcus epidermidis*	2	0.24
*Alcaligenes denitrificans*	1	0.12
*Bacteroides uniformis*	1	0.12
*Clostridium subterminale*	1	0.12
*Clostridium perfringens*	1	0.12
*Clostridium hastiforme*	1	0.12
*Corynebacterium amycolatum*	1	0.12
*Fusobacterium necrophorum*	1	0.12
*Gemella morbillorum*	1	0.12
*Lactobacillus paracasei*	1	0.12
*Pseudomonas oleovorans*	1	0.12
*Peptostreptococcus anaerobius*	1	0.12
*Peptostreptococcus prevotii*	1	0.12
*Peptostreptococcus tetradius*	1	0.12
*Prevotella loescheii*	1	0.12
*Prevotella oris*	1	0.12
*Providencia stuartii*	1	0.12
*Staphylococcus hominis*	1	0.12
*Staphylococcus lugdunensis*	1	0.12
*Staphylococcus simulans*	1	0.12
*Streptococcus* spp.	1	0.12

**Table 2:** Number of particular bacterial isolates in all patients with nonischemic diabetic foot syndrome.

	Number of isolates	Percent
*Staphylococcus aureus* MSS	88	14.47%
*Enterococcus faecalis*	48	7.89%
*Pseudomonas aeruginosa*	41	6.74%
*Enterobacter cloacae*	33	5.42%
*Escherichia coli*	32	5.26%
*Staphylococcus aureus* MRSA, MLSB	25	4.11%
*Acinetobacter baumannii* MDR	24	3.95%
*Klebsiella pneumoniae*	24	3.95%
*Enterobacter cloacae* ESBL	20	3.29%
*Proteus mirabilis*	17	2.80%
*Staphylococcus aureus* MSS, MLSB	17	2.80%
*Proteus* spp.	15	2.47%
*Enterococcus faecalis* HLAR	14	2.30%
*Klebsiella pneumoniae* ESBL	13	2.14%
*Streptococcus agalactiae*	12	1.97%
*Staphylococcus aureus* MRSA	12	1.97%
*Morganella morganii*	11	1.81%
*Acinetobacter baumannii*	10	1.64%
*Finegoldia magna*	10	1.64%
*Klebsiella oxytoca*	8	1.32%
*Enterobacter aerogenes*	8	1.32%
*Streptococcus mitis*	6	0.99%
*Stenotrophomonas maltophilia*	5	0.82%
*Peptoniphilus asaccharolyticus*	5	0.82%
*Enterococcus faecium* HLAR	5	0.82%
*Enterococcus faecium*	5	0.82%
*Veillonella* spp.	4	0.66%
*Proteus penneri*	4	0.66%
*Escherichia coli* ESBL	4	0.66%
*Citrobacter freundii*	4	0.66%
*Anaerococcus prevotii*	4	0.66%
*Bacteroides fragilis*	4	0.66%
*Streptococcus agalactiae* MLSB	3	0.49%
*Serratia marcescens*	3	0.49%
*Providencia rettgeri*	3	0.49%
*Pseudomonas aeruginosa* MDR, MBL	3	0.49%
*Pseudomonas aeruginosa* MDR	3	0.49%
*Streptococcus constellatus*	2	0.33%
*Proteus vulgaris*	2	0.33%
*Propionibacterium acnes*	2	0.33%
*Prevotella* spp.	2	0.33%
*Prevotella melaninogenica*	2	0.33%
*Peptostreptococcus* spp.	2	0.33%
*Morganella morganii* ESBL	2	0.33%
*Enterococcus faecium* HLAR, VRE	2	0.33%
*Corynebacterium striatum*	2	0.33%
*Clostridium novyi*	2	0.33%
*Citrobacter braakii* AMP C	2	0.33%
*Bacteroides distasonis*	2	0.33%
*Acinetobacter lwoffii*	2	0.33%
*Citrobacter braakii*	2	0.33%
*Streptococcus pyogenes*	1	0.16%
*Staphylococcus simulans*	1	0.16%
*Staphylococcus lugdunensis* MLSB, MRS	1	0.16%
*Staphylococcus epidermidis* MRS	1	0.16%
*Staphylococcus epidermidis*	1	0.16%
*Pseudomonas oleovorans*	1	0.16%
*Proteus mirabilis* ESBL	1	0.16%
*Prevotella oris*	1	0.16%
*Prevotella loescheii*	1	0.16%
*Peptostreptococcus tetradius*	1	0.16%
*Peptostreptococcus prevotii*	1	0.16%
*Peptostreptococcus anaerobius*	1	0.16%
*Pseudomonas aeruginosa* MBL	1	0.16%
*Lactobacillus paracasei*	1	0.16%
*Lactobacillus fermentum*	1	0.16%
*Fusobacterium necrophorum*	1	0.16%
*Enterococcus faecalis* HLAR, VRE	1	0.16%
*Enterobacter cloacae* AMP C, ESBL	1	0.16%
*Enterobacter cloacae* AMP C	1	0.16%
*Corynebacterium amycolatum*	1	0.16%
*Clostridium perfringens*	1	0.16%
*Clostridium subterminale*	1	0.16%
*Clostridium sporogenes*	1	0.16%
*Clostridium innocuum*	1	0.16%
*Clostridium hastiforme*	1	0.16%
*Citrobacter youngae* AMP C	1	0.16%
*Citrobacter youngae*	1	0.16%
*Citrobacter koseri*	1	0.16%
*Citrobacter freundii* ESBL	1	0.16%
*Bifidobacterium* spp.	1	0.16%
*Bacteroides uniformis*	1	0.16%
*Alcaligenes denitrificans*	1	0.16%
*Staphylococcus hominis*	1	0.16%

Abbreviations: MSS: methicillin-susceptible Staphylococcus; MRSA: methicillin-resistant *Staphylococcus aureus*; MLSB: macrolide-lincosamide-streptogramin B resistance; MDR: multiple drug resistant; ESBL: extended spectrum beta-lactamase; HLAR: high-level aminoglycoside resistance; MBL: metallo-beta-lactamase; VRE: vancomycin-resistant enterococci; AMP C: AmpC beta-lactamases.

**Table 3:** Number of particular bacterial isolates in all patients with ischemic diabetic foot syndrome.

	Number of isolates	Percent
*Staphylococcus aureus* MSS	38	16.10%
*Enterococcus faecalis*	26	11.02%
*Acinetobacter baumannii* MDR	14	5.93%
*Proteus mirabilis*	13	5.51%
*Staphylococcus aureus* MLSB	13	5.51%
*Staphylococcus aureus* MRSA, MLSB	11	4.66%
*Pseudomonas aeruginosa*	10	4.24%
*Enterobacter cloacae*	9	3.81%
*Klebsiella pneumoniae*	9	3.81%
*Escherichia coli*	8	3.39%
*Enterococcus faecalis* HLAR	7	2.97%
*Staphylococcus aureus* MRSA	7	2.97%
*Streptococcus agalactiae*	7	2.97%
*Acinetobacter baumannii*	6	2.54%
*Morganella morganii*	4	1.69%
*Proteus* spp.	4	1.69%
*Streptococcus dysgalactiae*	4	1.69%
*Enterococcus faecium*	3	1.27%
*Streptococcus mitis*	3	1.27%
*Streptococcus pyogenes*	3	1.27%
*Actinomyces naeslundii*	2	0.85%
*Citrobacter koseri*	2	0.85%
*Clostridium sporogenes*	2	0.85%
*Finegoldia magna*	2	0.85%
*Klebsiella pneumoniae* ESBL	2	0.85%
*Proteus vulgaris*	2	0.85%
*Stenotrophomonas maltophilia*	2	0.85%
*Streptococcus agalactiae* MLSB	2	0.85%
*Streptococcus constellatus*	2	0.85%
*Veillonella* spp.	2	0.85%
*Anaerococcus prevotii*	1	0.42%
*Bifidobacterium* spp.	1	0.42%
*Clostridium innocuum*	1	0.42%
*Enterobacter cloacae* AMP C, ESBL	1	0.42%
*Enterobacter cloacae* ESBL	1	0.42%
*Enterococcus faecium* HLAR	1	0.42%
*Enterococcus faecium* HLAR, VRE	1	0.42%
*Enterobacter aerogenes*	1	0.42%
*Gemella morbillorum*	1	0.42%
*Klebsiella oxytoca*	1	0.42%
*Klebsiella pneumoniae* MBL MDR	1	0.42%
*Klebsiella pneumoniae* MDR	1	0.42%
*Lactobacillus fermentum*	1	0.42%
*Prevotella* spp.	1	0.42%
*Providencia stuartii* ESBL, AMP C	1	0.42%
*Streptococcus dysgalactiae* MLSB	1	0.42%
*Streptococcus* spp.	1	0.42%

Abbreviations: MSS: methicillin-susceptible Staphylococcus; MDR: multiple drug resistant; MLSB: macrolide-lincosamide-streptogramin B resistance; MRSA: methicillin-resistant *Staphylococcus aureus*; HLAR: high-level aminoglycoside resistance; ESBL: extended spectrum beta-lactamase; AMP C: AmpC beta-lactamases; VRE: vancomycin-resistant enterococci; MBL: metallo-beta-lactamase.

**Table 4:** Occurrence of particular resistance mechanisms in all analyzed bacterial strains.

Species and resistance mechanism	Percentage of isolated strains with the particular mechanism
*Acinetobacter baumannii* MDR	70.37%
*Staphylococcus aureus* MRSA	9.00%
*Staphylococcus aureus* MLSB	13.74%
*Staphylococcus aureus* MRSA, MLSB	17.06%
*Enterococcus faecalis* HLAR	21.88%
*Enterococcus faecalis* HLAR, VRE (no = 4)	4.17%
*Enterococcus faecium* HLAR, VRE (no = 3)	17.64%
*Enterobacter cloacae* ESBL	32.31%
*Enterobacter cloacae* ESBL, AMP C (no = 2)	3.08%
*Klebsiella pneumoniae* ESBL	30%
*Klebsiella pneumoniae* MBL, MDR (no = 1)	0.50%
*Escherichia coli* ESBL	9.10%
*Proteus mirabilis* ESBL (no = 1)	3.20%
*Morganella morganii* ESBL (no = 2)	11.76%
*Pseudomonas aeruginosa* MDR, MBL (no = 3)	5.17%

MDR: multiple drug resistant; MRSA: methicillin-resistant *Staphylococcus aureus*; MLSB: macrolide-lincosamide-streptogramin B resistance; HLAR: high-level aminoglycoside resistance; VRE: vancomycin-resistant enterococci; ESBL: extended spectrum beta-lactamase; AMP C: AmpC beta-lactamases; MBL: metallo-beta-lactamase.

**Table 5:** Susceptibility of bacterial strains to antibiotics in the entire study group, in people with or without PAD (peripheral arterial disease).

Antibacterial agent	Susceptibility in all patients	Susceptibility in patients with PAD	Susceptibility in patients without PAD	Statistical significance, *p*
*Penicillins and penicillins with beta-lactamase inhibitor*				
Penicillin G	23%	20%	28%	*p* = 0.01891
Ampicillin	27%	26%	30%	*p* = 0.18143
Amoxicillin	26%	25%	30%	*p* = 0.12352
Amoxicillin with clavulanate	53%	51%	61%	*p* = 0.00945
Piperacillin with tazobactam	57%	57%	59%	*p* = 0.58503
*Cephalosporins*				
Cephalexin	26%	24%	31%	*p* = 0.03479
Cephadroxyl				
Cefazolin				
Cefaclor				
Cefuroxime	35%	33%	39%	*p* = 0.16400
Ceftazidime	30%	33%	24%	*p* = 0.01237
Cefotaxime	48%	47%	50%	*p* = 0.38042
Ceftriaxone	49%	47%	51%	*p* = 0.31700
Cefixime	31%	32%	27%	*p* = 0.17967
Ceftybuten	30%	32%	27%	*p* = 0.19485
Cefepime	62%	62%	64%	*p* = 0.58677
Ceftalozane	37%	39%	32%	*p* = 0.06028
Ceftaroline	58%	56%	64%	*p* = 0.05635
*Monobactams*				
Aztreonam	29%	31%	22%	*p* = 0.00712
*Carbapenems*				
Meropenem	82%	83%	80%	*p* = 0.32769
Imipenem with cilastatin	79%	80%	76%	*p* = 0.12597
Ertapenem	79%	79%	81%	*p* = 0.41590
*Glycopeptides*				
Vancomycin	50%	46%	58%	*p* = 0.00197
Teicoplanin	50%	46%	59%	*p* = 0.00135
Dalbavancin	50%	47%	59%	*p* = 0.00155
*Lipopeptides*				
Daptomycin	48%	45%	57%	*p* = 0.00199
*Aminoglycosides*				
Gentamicin	65%	67%	63%	*p* = 0.28476
Amikacin	65%	66%	65%	*p* = 0.86581
Tobramycin	68%	68%	69%	*p* = 0.87520
*Tetracyclines*				
Doxycycline	40%	38%	44%	*p* = 0.08849
*Glycylcycline*				
Tigecycline	75%	53%	80%	*p* = 0.05511
*Macrolides*				
Erythromycin	9%	9%	11%	*p* = 0.34070
Clarithromycin	9%	8%	10%	*p* = 0.46091
Azithromycin				
*Lincosamides*				
Clindamycin	24%	22%	28%	*p* = 0.07191
*Oxazolidinones*				
Linezolid	47%	43%	56%	*p* = 0.00087
*Fluoroquinolones*				
Ciprofloxacin	35%	36%	29%	*p* = 0.10831
Levofloxacin	37%	39%	29%	*p* = 0.01410
Moxifloxacin	31%	34%	24%	*p* = 0.02502
*Sulfonamides*				
Cotrimoxazole	45%	45%	46%	*p* = 0.78042
*Nitroimidazoles*				
Metronidazole	4%	5%	2%	*p* = 0.02121
*Polymyxins*				
Colistin	34%	38%	26%	*p* = 0.00155

**Table 6:** Susceptibility of isolates to the most commonly recommended combinations of antibacterial agents in the literature in patients with and without peripheral arterial disease (PAD).

Antibacterial agents	Susceptibility in all patients	Susceptibility in patients with PAD	Susceptibility in patients without PAD	Statistical significance, *p*
Ciprofloxacin with clindamycin	46%	45%	46%	*p* = 0.90248
Levofloxacin with clindamycin	44%	45%	40%	*p* = 0.30085
Amoxicillin/clavulanate with vancomycin	62%	58%	70%	*p* = 0.00109
Piperacillin/tazobactam with vancomycin	87%	85%	94%	*p* = 0.00050
Imipenem with vancomycin	88%	89%	85%	*p* = 0.13444
Meropenem with vancomycin	91%	91%	89%	*p* = 0.36788
Amoxicillin/clavulanate with cotrimoxazole	73%	71%	79%	*p* = 0.01562
Ceftazidime with metronidazole	33%	36%	24%	*p* = 0.00178
Ceftazidime with clindamycin	52%	53%	49%	*p* = 0.27473
Ciprofloxacin with linezolid	64%	61%	69%	*p* = 0.07458
Moxifloxacin with linezolid	59%	58%	63%	*p* = 0.28648

## Data Availability

Data available on request; please contact Rafał Małecki, e-mail: rafal.malecki@umed.wroc.pl.
